# Mechanical, thermal, structure and radiation shielding efficiency of natural kaolinite-based composites reinforced with heavy metal oxides

**DOI:** 10.1038/s41598-026-40686-1

**Published:** 2026-03-17

**Authors:** Mohamed. Elsafi, Samer E’layan Alawaideh, Mohamed A. Hamada, M. I Sayyed

**Affiliations:** 1 Physics Department, Faculty of Science, Alexandria University, Alexandria, 21511 Egypt; 2 Department of Chemistry, Faculty of Science, Isra University, Amman, Jordan; 3 Materials Science Department, Institute of Graduate Studies and Research, Alexandria University, Alexandria, Egypt; 4 Department of Physics, Faculty of Science, Isra University, Amman, Jordan

**Keywords:** Kaolinite, Heavy metal oxide, Thermal stability, Radiation shielding, Building materials, Engineering, Materials science

## Abstract

In this work, a low-cost, natural kaolinite-based matrix reinforced with gypsum and ground marble waste was prepared with the aim of developing materials suitable for radiation shielding applications. The prepared matrix was reinforced with a fixed 30 wt% of various metal oxides, including bismuth oxide (Bi_2_O_3_), tungsten oxide (WO_3_), copper oxide (CuO), iron oxide (Fe_2_O_3_), and titanium oxide (TiO_2_). The prepared clay composites were characterized using X-ray diffraction (XRD) to determine the crystalline phases, Fourier transform infrared (FTIR) spectroscopy to study the chemical bonds, and Scanning electron microscope (SEM) to evaluate the surface morphology and the distribution of the additives within the matrix. The mechanical and thermal properties of the composites were also evaluated, along with their efficiency in attenuating gamma rays at different energies. The results showed that reinforcing the natural matrix with different oxides led to a significant improvement in density and thermal stability, as well as a marked increase in radiation attenuation coefficients compared to the unreinforced matrix. The shielding efficiencies for a 3 cm thickness were 35.14, 38.16, 39.67, 40.67, 43.33 and 45.51% for Reference, C-Ti, C-Fe, C-Cu, C-W and C-Bi, respectively. The clay sample reinforced with CuO exhibited the highest mechanical strength, while the clay sample reinforced with Bi_2_O_3_ exhibited the highest shielding efficiency due to the high density. These results confirm the potential use of the proposed composites as environmentally friendly and low-cost building materials for radiation shielding applications.

## Introduction

 In different scientific fields and technological applications, metal oxides are classified as one of the most extensively used materials. These metal oxides are well-known owing to their availability and common uses in a considerable range of industries such as solar cells, photonic devices, glasses, dielectric layers, thermoelectric devices, supercapacitors and batteries, gas sensing devices and radiation shielding materials^1–4^.

Given the real danger of ionization radiation for humans and environment, radiation protection is a noteworthy field of focus for several research groups. Suitable shielding products are used in different industrial and nuclear power plants, research laboratories, nuclear waste storage, medical facilities and research reactors. Several previous works studied glasses, ceramics, alloys, nanocomposites and clay as effective products for this purpose^5–8^.

Clay is an economical structure material which frequently utilized in construction and building. The attractive features of clay-based products like strength, durability, availability and ecofriendly implies that there is still great need for the clay in different fields, despite the commercially available of advanced alternative materials such as plastics, steel, glass and concrete.

Clays based radiation shields are receiving increasing attention as suitable radiation shielding product because of their interesting characteristics. For instance, clay is considered as low-cost materials, easy to mold and shape, environmentally friendly and safe, and can mix with heavy metal oxides. All these interesting properties encourage the researchers to use clay with several additives to design novel radiation shielding products^9,10^.

The incorporation of heavy metal oxides is a common technique to enhance the radiation shielding effectiveness of the clay, since the density plays a major role in the photon-matter interaction. Lead (Pb) is widely used as additives to improve the radiation shielding effectiveness of the clay. Unfortunately, continued exposure to Pb has a negative impact on the humans and causes some diseases such as cancer. Also, lead has some limitations including heaviness in weight and low chemical stability. The aforementioned limitations have driven radiation shielding engineers to seek safer additives such as Bi_2_O_3_, WO_3_, CuO…etc^11,12^.

Such metal oxides are utilized in radiation shielding to offer some benefits such as eco-friendliness, cost effectiveness, structural strength and enhanced attenuation. Among the leading desirable metal oxides in radiation shielding is bismuth oxide as one of the primary options to replace PbO. In comparison to PbO, bismuth oxide is non-hazard and the utilization of Bi_2_O_3_ as an additive to clay can considerably improve the density of the net composite. Moreover, Bi_2_O_3_ enhances the mechanical strength of clay, improves the chemical and thermal stability^13^.

Tungsten (VI) oxide (WO_3_) is another primary option to replace PbO in radiation shielding technology. WO_3_ has some distinct features and the addition of WO_3_ to the clay can provide superior hardness, improve chemical stability, reduce porosity and reduces the transmission of photons through the clay^14^.

To properly analysis the radiation shielding features of shielding materials, multiple parameters must be carefully measured. The mass attenuation coefficient is one of these key quantities, allowing the determination of different related factors such as the half value layer and radiation shielding efficiency^15–19^. The narrow beam geometry is a standard technique commonly employed to measure the radiation shielding parameters of the materials. This procedure mainly requires detector system gamma ray sources, lead collimator…etc. Cs-137, Co-60 and Am-241 are the main sources used in different radiation shielding study^20^. This is due to some reasons such as they are suitable for low and high energy applications, allow comparison with previously available data, reliable for testing detector effectiveness, and offer long half-life for repeated tests.

In this work, we developed eco-friendly, low-cost clay composites as building materials for use as shielding walls against ionizing radiation. These composites are based on kaolinite clay with the addition of ground marble residue, gypsum, and a percentage of some heavy oxides. The mechanical, thermal, and structural properties of the prepared clay composites were studied, along with their attenuation properties at various gamma energies, to determine their shielding effectiveness against gamma and X-ray radiation.

## Materials and methods

### Clay composites preparation

First, the clay material (kaolinite) was collected and air-dried to remove any excess moisture. It was then ground using a suitable mill until a fine powder was obtained. Next, the powder was sieved using a 180-$\:\mu\:m$ sieve to ensure uniform particle size and distribution. Similarly, some marble remnants were collected, ground, and sieved using the same sieve to achieve a particle size similar to the other components. Clay samples were prepared in fixed weight ratios, with each sample also weighing 18 g. The clay composition consisted of 70% as a matrix (60% kaolinite, 20% gypsum, and 20% waste marble) and 30% various heavy metal oxides as supporting additives, as shown in Table 1.

**Table 1:** The codes and compositions of prepared advanced clay-composites.

Code	Composition, wt%								Bulk Density, g.cm^− 3^	Open Porosity, %
	Kaolinite	Gypsum	Marble	TiO_2_	Fe_2_O_3_	CuO	WO_3_	Bi_2_O_3_		
Reference	60	20	20	−	−	−	−	−	1.775	29.3
C-Ti	42	14	14	30	−	−	−	−	1.983	30.9
C-Fe	42	14	14	−	30	−	−	−	2.108	30.3
C-Cu	42	14	14	−	−	30	−	−	2.196	29.5
C-W	42	14	14	−	−	−	30	−	2.209	30.3
C-Bi	42	14	14	−	−	−	−	30	2.227	32.1

**Fig. 1:**
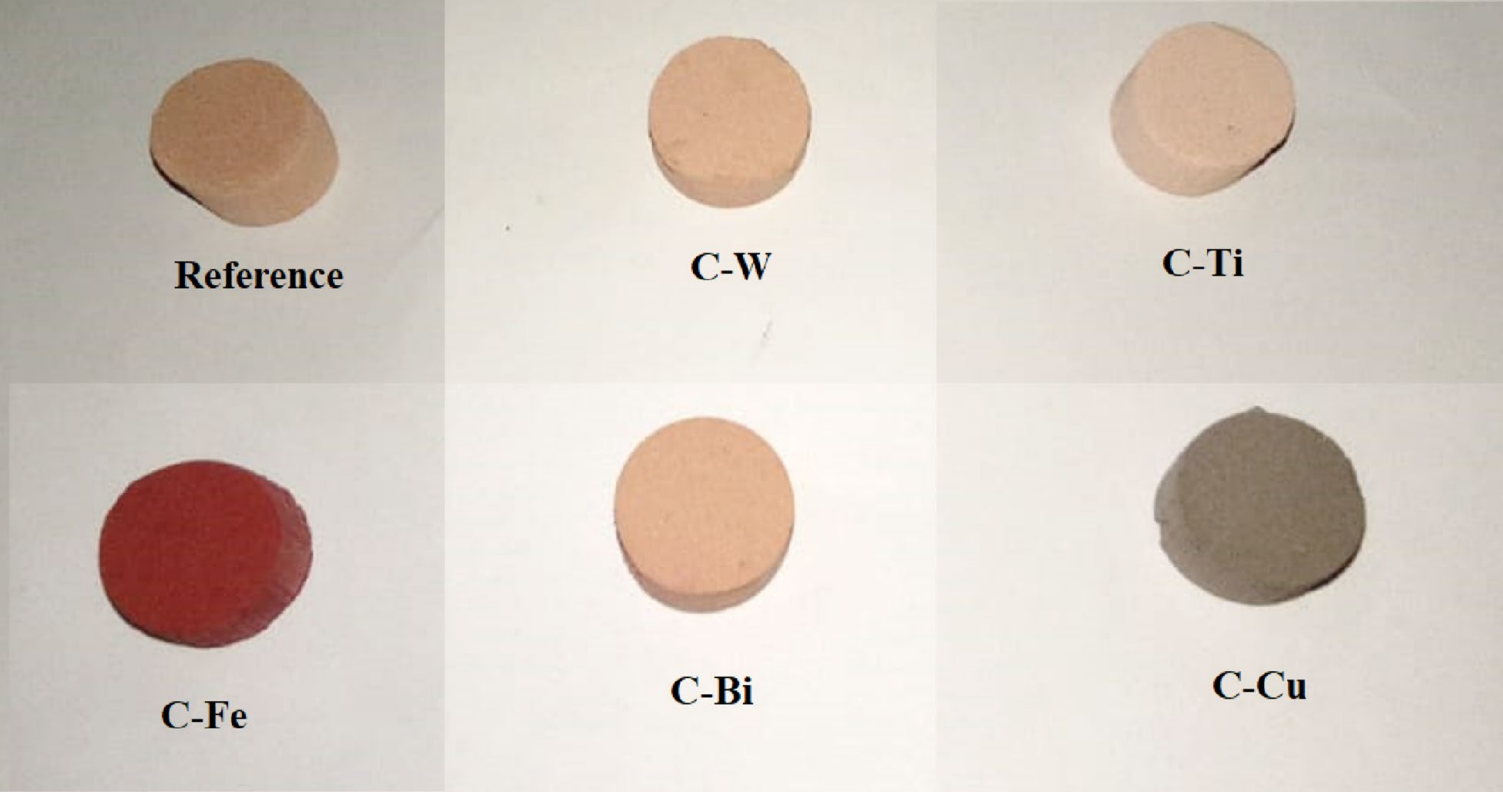
Picture of prepared samples.

The dry powders were thoroughly mixed and kneaded for a sufficient time to achieve a homogeneous mixture free of aggregation, and then placed in cylindrical molds. The samples were then left to air dry at room temperature for a sufficient period. After that, the samples were placed in an electric oven and subjected to gradual heating. They were first heated at 50 °C for 20 h to slowly remove any remaining moisture within the structure. The temperature was then increased to 110 °C for 3 h, followed by natural cooling within the oven until they reached room temperature. After the initial heat treatment, the samples were dimensional standardization and removal of any potential surface defects using sandpaper, thus improving the accuracy of experimental measurements. The samples then subjected to a second heating process, being heated at 650 °C for 2 h, and were then allowed to cool gradually to room temperature. The final picture of prepared clay composites is shown in Fig. 1.

### Clay characterization

The bulk density ($\:\rho\:$)and open porosity ($\:OP$) of advanced clay samples was measured using Archimedes principle^21,22^, since the clay composite was weighed dry (w_d_), wet (w_w_), and suspended(w_s_) in distilled water. The bulk density and porosity were calculated from the following relationships:

1 $$\:\rho\:=\frac{{w}_{d}}{{w}_{w}-{w}_{s}}$$

2 $$\:OP,\:\%=\frac{{w}_{w}-{w}_{d}}{{w}_{w}-{w}_{s}}\times\:100$$

Fourier transform infrared spectrometry (FTIR spectrometer, (Model: Bruker Tensor 37)) was used to study the chemical bonds present in the prepared samples.

X-ray diffraction analysis (X-ray diffractometer, Model: Bruker XRD D2 phaser) was performed to verify the structural nature of the prepared clay samples and to study the phases formed after heat treatment.

The mechanical properties of the clay samples were evaluated by compressive strength testing using a Universal Testing Machine (Model: Tinius Olsen 5ST). The results reflected the influence of chemical composition on the mechanical performance.

The thermal properties of the samples were studied using a thermal analyzer (Model: Q600 thermogravimetric analyzer, TA instruments) to assess the thermal stability and behavior of the samples at high temperatures.

Scanning electron microscopy (SEM, model: model: FEI Quanta FEG 250) was used to study the surface morphology and cross-section, as well as the particle distribution, of the prepared clay samples. The SEM images helped assess the degree of structural homogeneity.

**Fig. 2:**
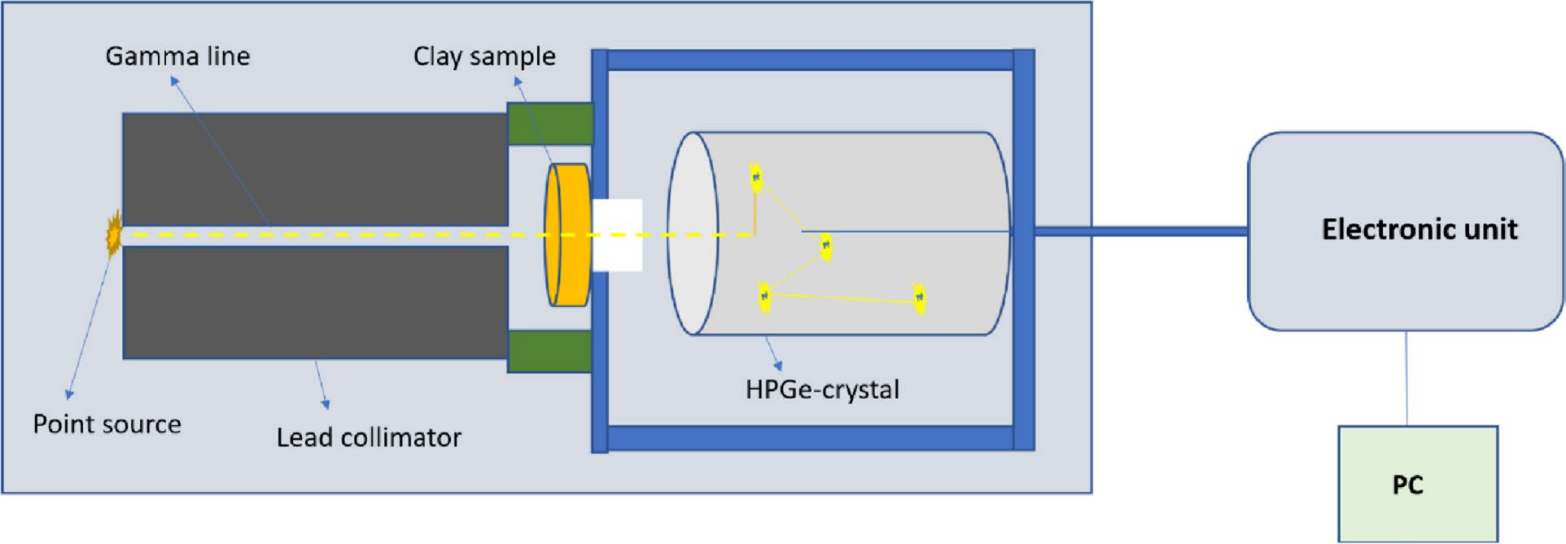
Geometry of attenuation measurements.

The shielding capacity of the prepared clay samples against ionizing photons (gamma rays and X-rays) was tested. The study was conducted experimentally using three gamma-ray point sources and a gamma detector. The sources were Am-241 (0.059 MeV line), Cs-137 (0.662 MeV line), and Co-60 (1.173 & 1.333 MeV two lines). The gamma detector used was a high-pure germanium (HPGe) detector with a relative efficiency of 24% and a resolution of 1.96 keV at the highest cobalt energy^23^. The mechanism of measurement used is shown in Fig. 2. The source and detector were positioned axially with15 cm distance in between. A lead collimator (10 cm long with an internal diameter of 0.8 cm) was placed between them, with the source on top of the collimator. In this case, the detector was first celebrated and then runs for a suitable time for each source to obtain the lowest measurement error. The measurement produced peaks according to the incident energies. These peaks have an intensity that is determined and called the initial intensity (Io). Subsequently, the clay sample to be measured was added below the collimator (in between the collimator and the detector), and the intensity was determined again after a suitable measurement time. In this case, it is called the transmitted intensity (I). From The Thickness of clay sample (x) and these intensities, the probability of gamma-ray interaction with clay sample through certain distance or call linear attenuation coefficient (LAC) can be determined by the following Eqs^24–26^. :

3 $$\:LAC\:=\:\frac{1}{x}\:\mathrm{l}\mathrm{n}\left(\frac{\mathrm{I}\mathrm{o}}{\mathrm{I}}\right)$$

From LAC values, other parameters can be measured using the following Eqs^27–30^.

4 $$\:HVL\:=\:\frac{\mathrm{ln}\left(2\right)}{LAC}$$

5 $$\:TVL\:=\:\frac{\mathrm{ln}\left(10\right)}{LAC}$$

6 $$\:RSE,\%\:=\:(1\:-\:\frac{\mathrm{I}}{\mathrm{I}\mathrm{o}})\:*\:100$$

## Results and discussion

### Clay density

**Fig. 3:**
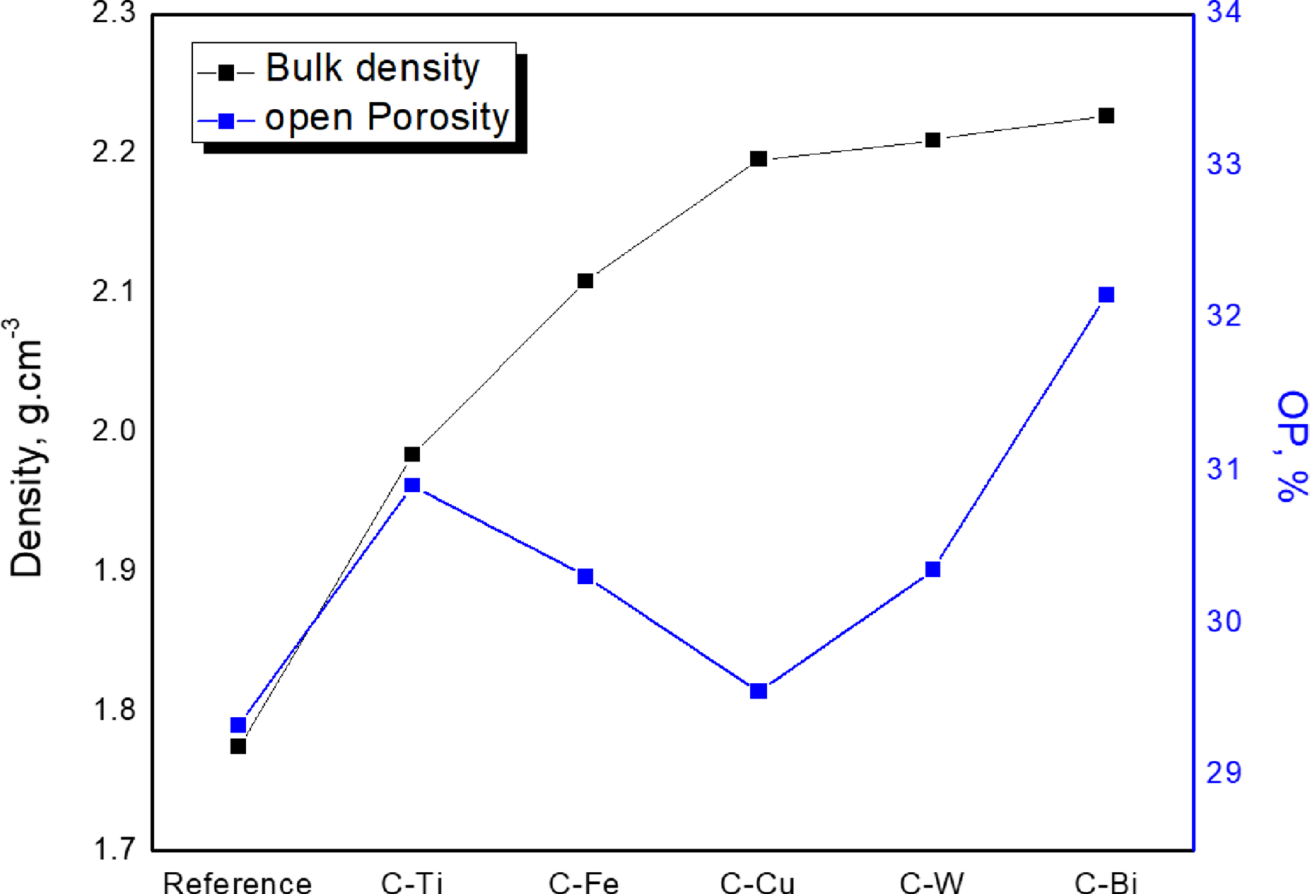
Density of advanced clay samples.

Figure 3 shows the BD, g.cm^− 3^ and porosity (P, %) of the prepared clay samples. The BD’s ranged from 1.775 g.cm^− 3^ for the reference clay samples to 2.227 g.cm^− 3^ for clay including Bi_2_O_3_ (C-Bi) with 19.4% increased percentage due to the addition of HMO. On other hand, the highest open porosity was for C-Bi composite while the lowest value was for C-Cu as shown in Fig. 3; Table 1. The higher open porosity of C-Bi is likely related to weaker bonding and poor packing, while C-Cu exhibits better densification and therefore lower porosity.

### FTIR spectroscopy

**Fig. 4:**
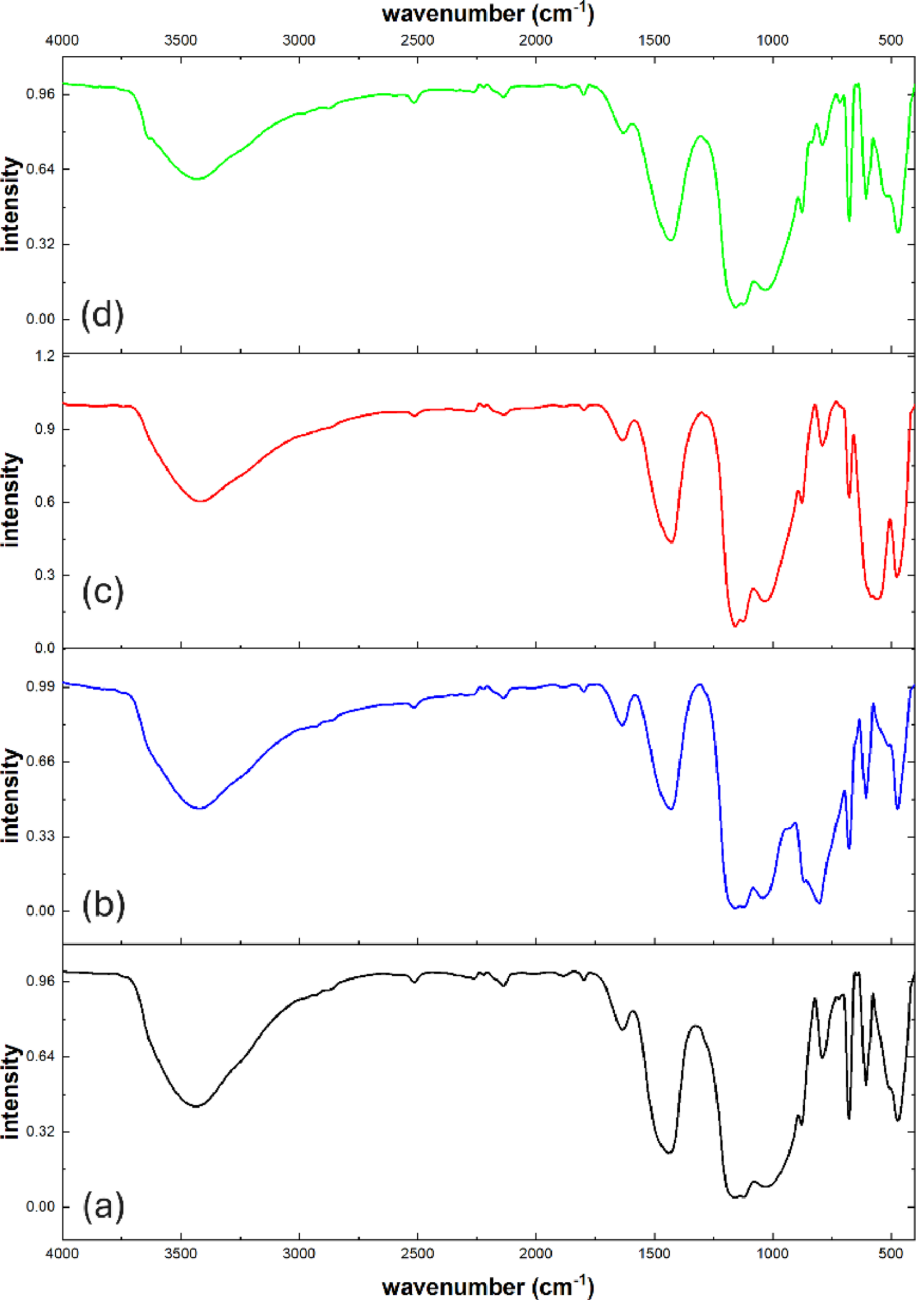
IR spectra of (**a**) Reference, (**b**) C-W, (**c**) C-Fe, and (**d**) C-Bi samples.

Figure 4 shows IR spectra for reference sample and the samples containing WO_3_, Fe_2_O_3_, and Bi_2_O_3_ (C-W, C-Fe, C-Bi, respectively). It is observed in Fig. 4a the appearance of peaks at 3435 and 1635 cm^− 1^ which are related to stretching and bending vibrations of adsorbed water. Also, the peaks at 1031, 788, and 470 cm^− 1^ which corresponding to Si-O bond vibration, Si-O-Si stretching and bending vibration, respectively^31–33^. In addition, the peaks at 1164 and 1122 cm^− 1^ are attributed to stretching vibrations in (SO_4_)^2−^ ions. Furthermore, the peaks at 678 and 605 cm^− 1^ are related to bending vibrations in (SO_4_)^2−^ ions^34,35^. As well, the peaks at 1440 and 879 cm^− 1^ which are related to stretching and bending vibrations in (CO_3_)^2−^ ions^36,37^. Also, the appearance of peaks at 2515 and 1799 cm^− 1^ that are related to calcite mineral^38,39^. All of these peaks confirm the prescence of three components in reference sample. On other hand, the new peak appeared in Fig. 4b at 806 cm^− 1^ which corresponding to W-O-W vibration^40,41^. Also, the new peak appeared in Fig. 4c at 559 cm^− 1^ that is related to Fe-O bond vibration^42,43^. In Fig. 4d, new peak appeared at 518 cm^− 1^ which is attributed to Bi-O stretching vibration^44^. The appearance of these new peaks confirms presence of different metal oxides in samples C-W, C-Fe, and C-Bi.

### X-ray diffraction

**Fig. 5:**
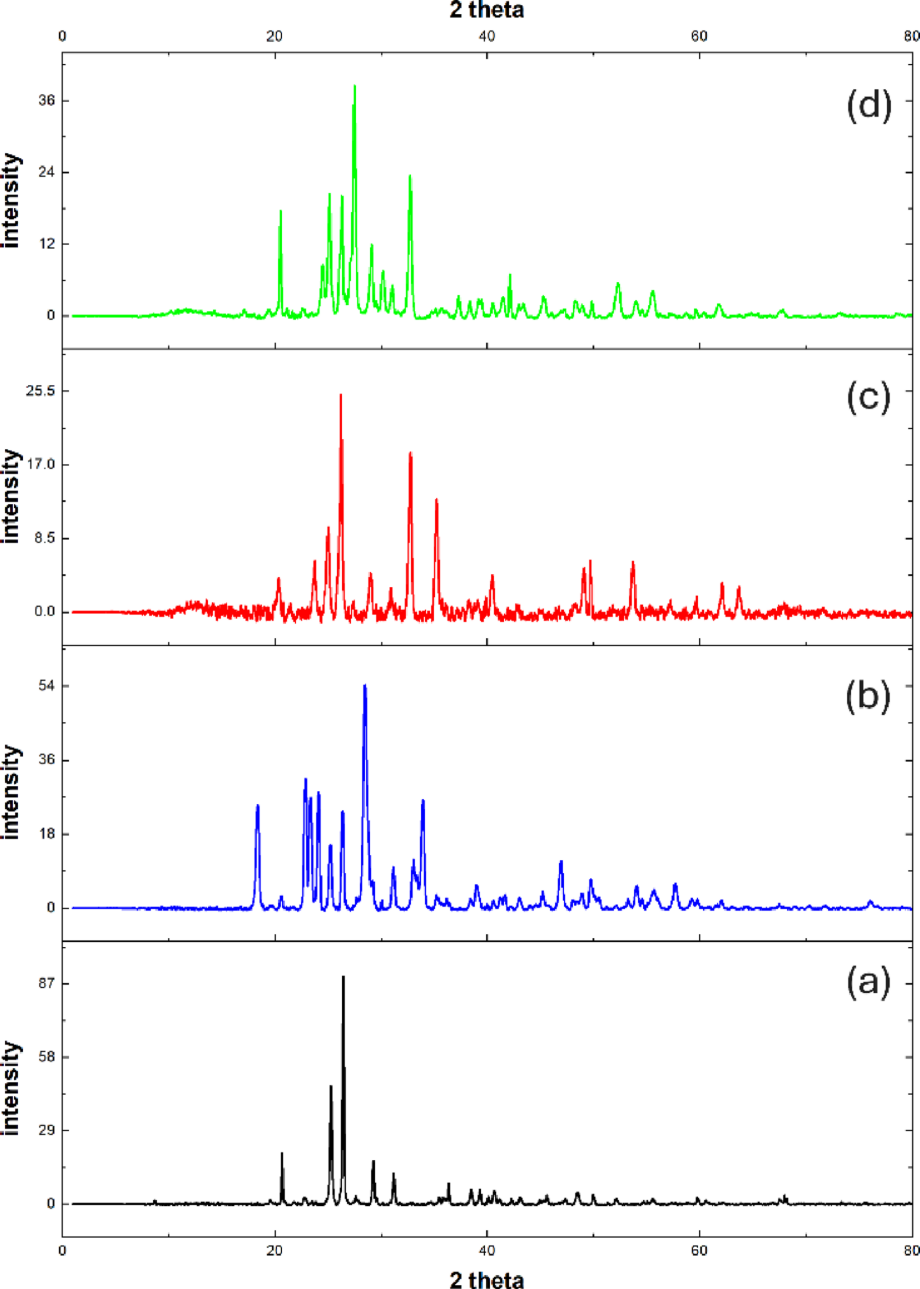
XRD patterns of (**a**) Reference, (**b**) C-W, (**c**) C-Fe, and (**d**) C-Bi samples.

Figure 5 shows XRD patterns for reference sample and the samples containing WO_3_, Fe_2_O_3_, and Bi_2_O_3_ (C-W, C-Fe, C-Bi, respectively). Figure 5a exhibits several diffraction peaks such as 20.68° and 26.44° which are related to quartz^39,45^. Also, appearance of diffraction peaks at 25.27°, 31.18°, 38.48°, and 39.30° which corresponding to gypsum^46^. Also, it is observed presence of diffraction peaks at 29.25°, 36.35°, and 48.46°. these peaks are attributed to calcite which is main component of marble^39,47^. In Fig. 5b, it is noticed appearance of new peaks related to WO_3_ at 18.38°, 22.88°, 23.35°, 24.11°, 28.49°, 33.09°, and 33.93°^48,49^. while Fig. 5c confirms presence of Fe_2_O_3_ in the C-Fe sample where the peaks at 23.71°, 32.76°, 35.25°, 49.68°, and 53.69° are related to Fe_2_O_3_^43,50^. On other hand, the peaks appeared in Fig. 5d at 24.49°, 27.48°, 30.16°, and 32.72° are related to Bi_2_O_3_^51,52^. The results of XRD are compatible with IR results which confirm addition of metal oxides into different samples.

### Thermal gravimetric analysis (TGA)

**Fig. 6:**
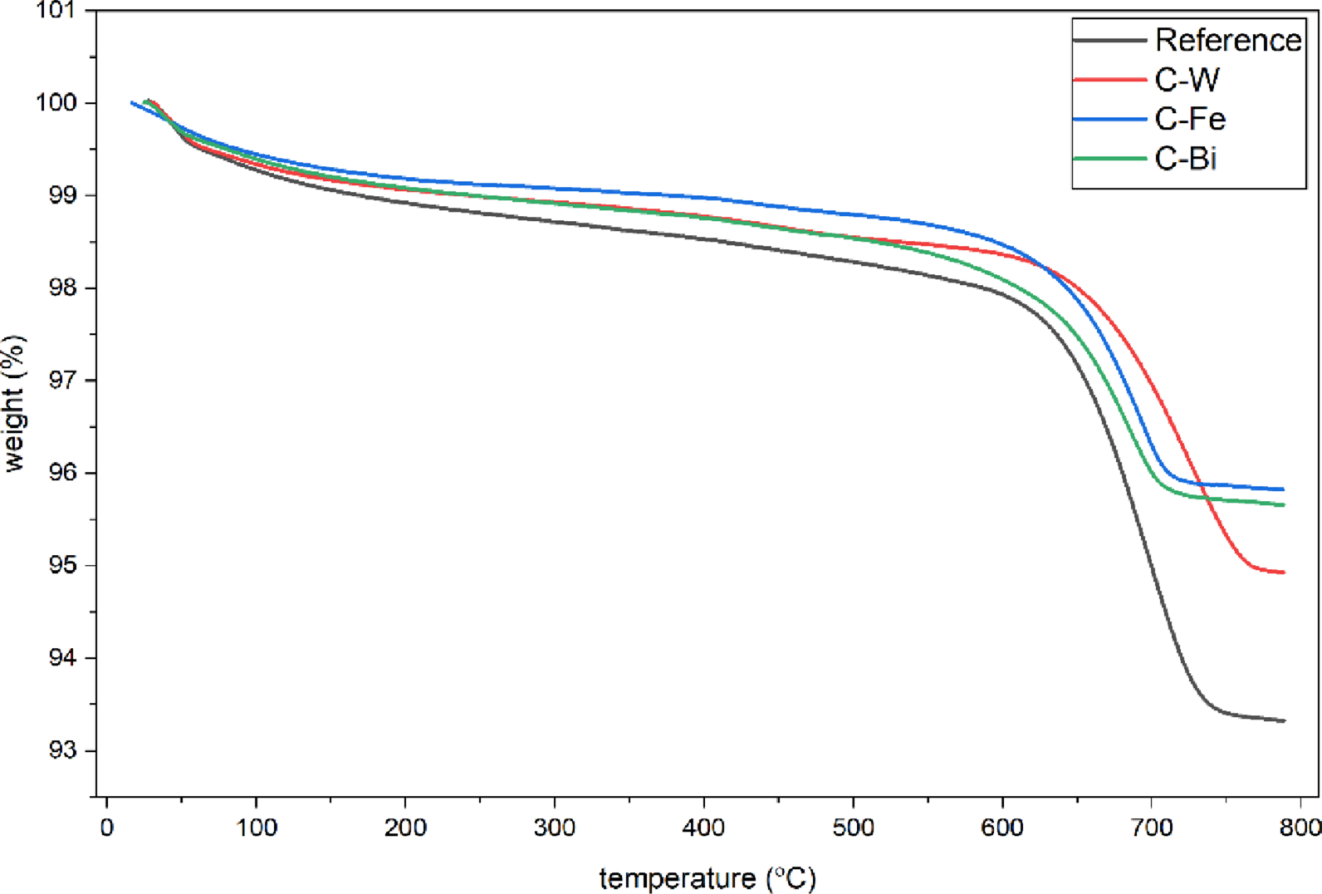
TGA curves of Reference, C-W, C-Fe, and C-Bi samples.

Figure 6 shows TGA curves of Reference, C-W, C-Fe, and C-Bi samples. Reference sample exhibits the weight loss of 6.67% with two thermal degradation steps. The first with a weight loss of 1.19% is related to evaporation of adsorbed water. The second with a weight loss of 5.44% is attributed to dihydroxylation of silicate structure in clays and dehydration of gypsum^53,54^. The other samples (C-W, C-Fe, and C-Bi) show the same thermal degradation stages with different rates. It is noticed increasing of residual mass with addition of different metal oxides where the C-W, C-Fe, and C-Bi samples exhibit weight loss of 5.07%, 4.18%, and 4.34%, respectively. These results confirm improvement of thermal stability with addition of WO_3_, Fe_2_O_3_, and Bi_2_O_3_.

### Compressive strength test

**Fig. 7:**
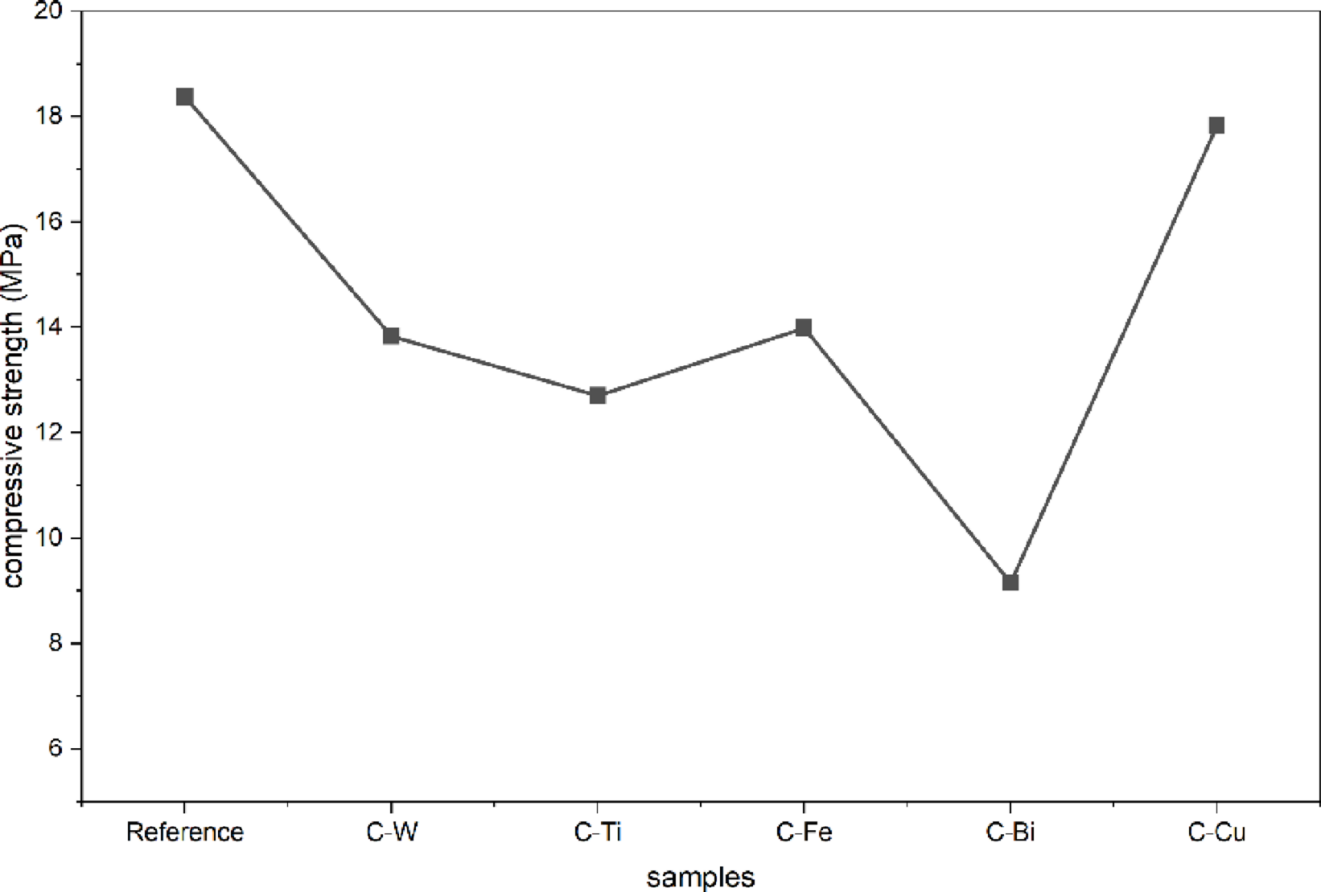
Compressive strength values of different samples.

Figure 7 shows compressive strength values of Reference (C-0), C-W, C-Ti, C-Fe, C-Bi, and C-Cu samples. The reference sample shows highest compressive strength value of 18.41 MPa and it decreases with addition of different metal oxides which may be attributed to agglomeration of metal oxides particles that makes stress concentration sites in samples and weak bonding between components of samples and the metal oxides. On other hand, the samples C-W, C-Ti, C-Fe, C-Bi, and C-Cu have compressive strength values of 13.4, 12.7, 14.0, 9.1, 17.8 MPa, respectively where the sample containing CuO (C-Cu) exhibits highest value while the sample containing Bi_2_O_3_ (C-Bi) exhibits lowest value due to the effect of porosity as well.

### Scanning electron microscope

**Fig. 8:**
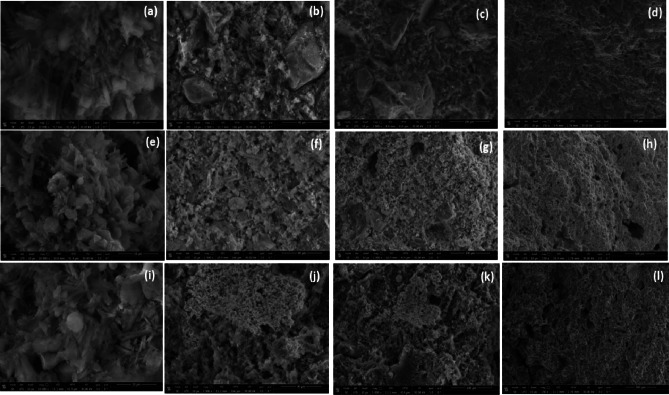
SEM images of (**a**-**d**) Reference, (**e**-**h**) C-W, and (**i**-**l**) C-Bi samples.

Figure 8 shows SEM images of Reference, C-W, and C-Bi samples at different magnifications. All SEM images show needle like crystals which are related to gypsum^55^. Also, stacked thin plates and irregular particles appeared which may be attributed to clay minerals. In addition, SEM images of C-W and C-Bi samples which contain metal oxides show dispersed particles that are related to metal oxides. On other hand, SEM images show many voids which affect negatively on mechanical properties of samples.

### Radiation shielding properties

This part exhibits the results of the radiation attenuation measurements and investigates the effect of altering the types of oxide in the prepared clay samples. In this study, experimental determination of attenuation factors has been conducted at four energy levels. From the obtained data, one also can study the effect of thickness of the fabricated clays on the attenuation factors (especially the TF and RPE). It is essential to keep in mind that the type of the oxide used into the clay has a major impact on the density of the net composite and from this we can understand the different in the attenuation factors between the different composites.

**Fig. 9:**
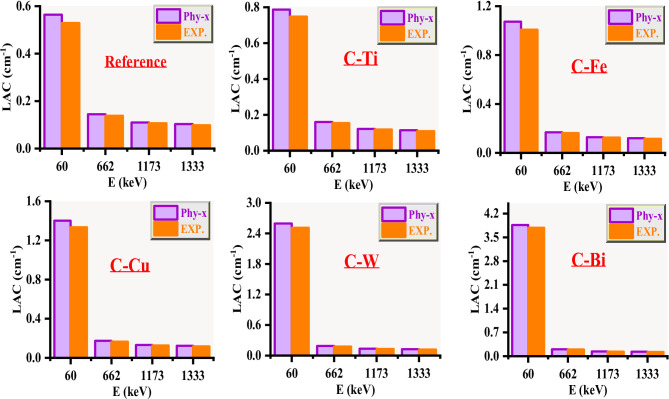
Comparison between the LAC values (both the measured and Phy-X) for the pure and modified clays.

Firstly, the LAC for the clay composites was measured between 0.059 and 1.333 MeV and was also calculated using Phy-X for validation purposes. A comparison between the LAC values (both the measured and Phy-X) are exhibited in Fig. 9. The difference between both approaches is found in the range of 2.85–6.65% for the reference sample (i.e. C-0), 2.81–5.22% for C-Ti, 2.41–6.50% for C-Fe, 4.41–5.02% for C-Cu, 3.34–5.97% for C-W and 2.14–4.95% for C-Bi. This relatively small difference indicates that it is possible to estimate the LAC data with the experimental setup used in this work.

**Fig. 10:**
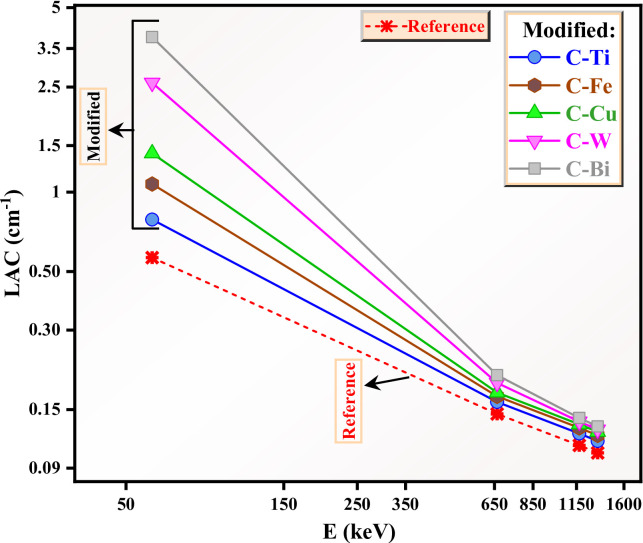
The linear attenuation coefficient for the prepared clays.

The effect of the oxides used in the prepared clays is discussed in Fig. 10. First observation from Fig. 10, all the clays with certain oxide has higher LAC than the reference sample, indicating an enhancement in the attenuation factor due to the addition of the oxides. Moreover, C-Bi has the highest LAC value among all the clay samples, which is as a result of the presence of Bi_2_O_3_ in this clay. Also, the density of this clay is higher than the other samples, so we can see the high difference LAC values between this clay and the reference clay. For example, the LAC for the reference clay at 0.059 MeV is 0.564 cm^− 1^, increasing to 3.864 cm^− 1^ due to the addition of Bi_2_O_3_. The LAC for the clays follows the order: C-Bi > C-W > C-Cu > C-Fe > C-Ti > C-0. This corresponds to the arrangement of clay density, such that the clay with the highest density has the highest LAC, and vice versa. At 0.662 MeV, the clays follow the same order (i.e. C-Bi has the maximum LAC, while the minimum LAC was reported for the reference clay). The same observation is reported at other energies, indicating a direct relationship between the density and the LAC of the clays. We compared the LAC of the reference clay to that of the clays with oxides at 0.059 MeV. An enhancement in the LAC is found by 28% due to the addition of TiO_2_, while LAC increases by 47% for C-Fe compared to the reference clay. When compare the LAC of the reference sample with C-Bi at 0.059 MeV, we found that the LAC increased by 85%. At 662 keV, the LAC is enhanced by 10%, 14%, 17%, 24% and 29% due to the addition of TiO_2_, Fe_2_O_3_, CuO, WO_3_ and Bi_2_O_3_ to the reference clay.

**Fig. 11:**
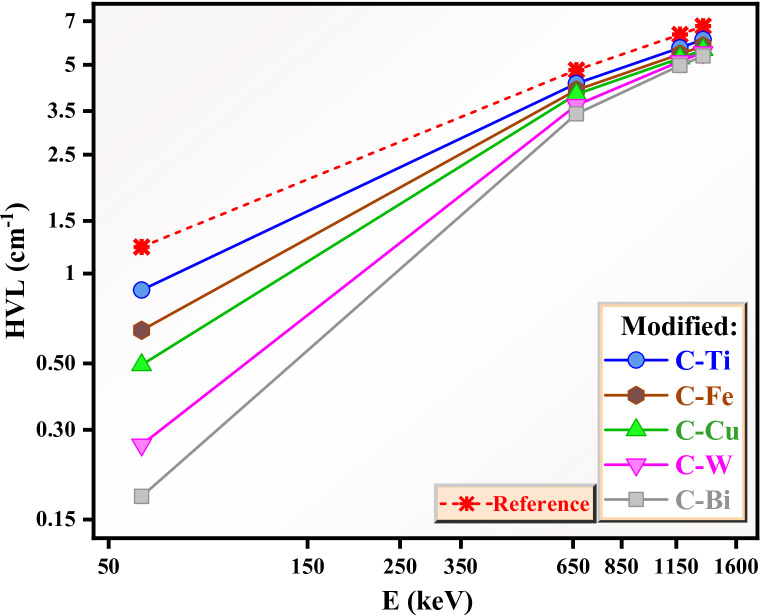
The half value layer for the prepared clays.

The HVL for the reference clay and the clay samples with the additives is plotted in Fig. 11. among the fabricated clays, the reference clay shows the highest HVL, followed by C-Ti. This implies low shielding performance of both reference clay and the clay with TiO_2_. This inclusion of other oxides reduced the HVL suggesting an enhancement in the shielding performance. Both clay with WO_3_ and Bi_2_O_3_ show low HVL comparing to other clays, indicating their superior shielding capability. This is due to the high Z for both elements W and Bi which increase the photon absorption via photoelectric effect (at 0.059 MeV) and Compton scattering (for E > 0.662 MeV). The high HVL for the C-Ti clay comparing to HVL for the other clays with different oxides indicates a weak attenuation ability of this clay and this occur sine the TiO_2_ has a small density. The clays with Fe_2_O_3_ and CuO have intermediate HVL, indicating moderate enhancement in shielding effectiveness. Accordingly, the observed decrease in the HVL for the clays with incorporating the different oxides implies that these oxides strengthen gamma radiation interactions chances and improves the radiation shielding performance. On the other hand, for all clays, the HVL shows a positive correlation with the energy. The lowest HVL is observed at 0.059 MeV, suggesting that lower energy radiation is less penetrating and hence needed less thickness of clay sample for attenuation. The HVL at 0.662 MeV is 4.803 cm for C-0 or Reference sample, while it is varied between 3.425 and 4.327 to cm for the clays with the difference additives. At 1.333 MeV, the HVL for C-0 increases to 6.742 cm, and varied between 5.353 and 6.079 cm for the clays with different oxides.

**Fig. 12:**
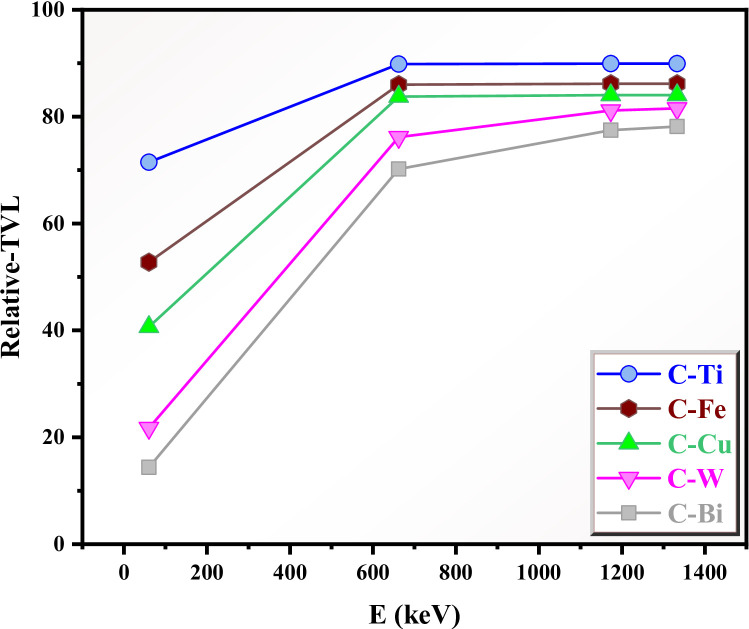
The relative tenth value layer (relative-TVL) for the clays.

We calculated another parameter called relative tenth value layer (relative-TVL) which represents the ratio between the TVL for the modified clay to the TVL of the reference clay. If the relative-TVL is 100%, then no improvement in the radiation shielding is occurred for the modified clay, since the TVL values for the modified and reference clay are the same. If the relative-TVL < 100%: then the radiation shielding for the modified clay is enhanced, and it has shorter TVL than the reference clay. The results of the + relative-TVL is presented in Fig. 12. Clearly, at the photoelectric effect (i.e. 0.059 MeV), all modified clays show small relative-TVL values (especially C-W and C-Bi), implying strong radiation shielding. At 0.662 MeV, the relative-TVL increases and ranging from 70% to 89%, and the relative-TVL converges which means that the atomic number of the different oxides is weakly influence the radiation shielding, since the Compton scattering dominates at this energy level. From Fig. 12, C-W and C-Bi have lower relative-TVL than the other modified clays, confirming better attenuation effectiveness of these two composites.

**Fig. 13:**
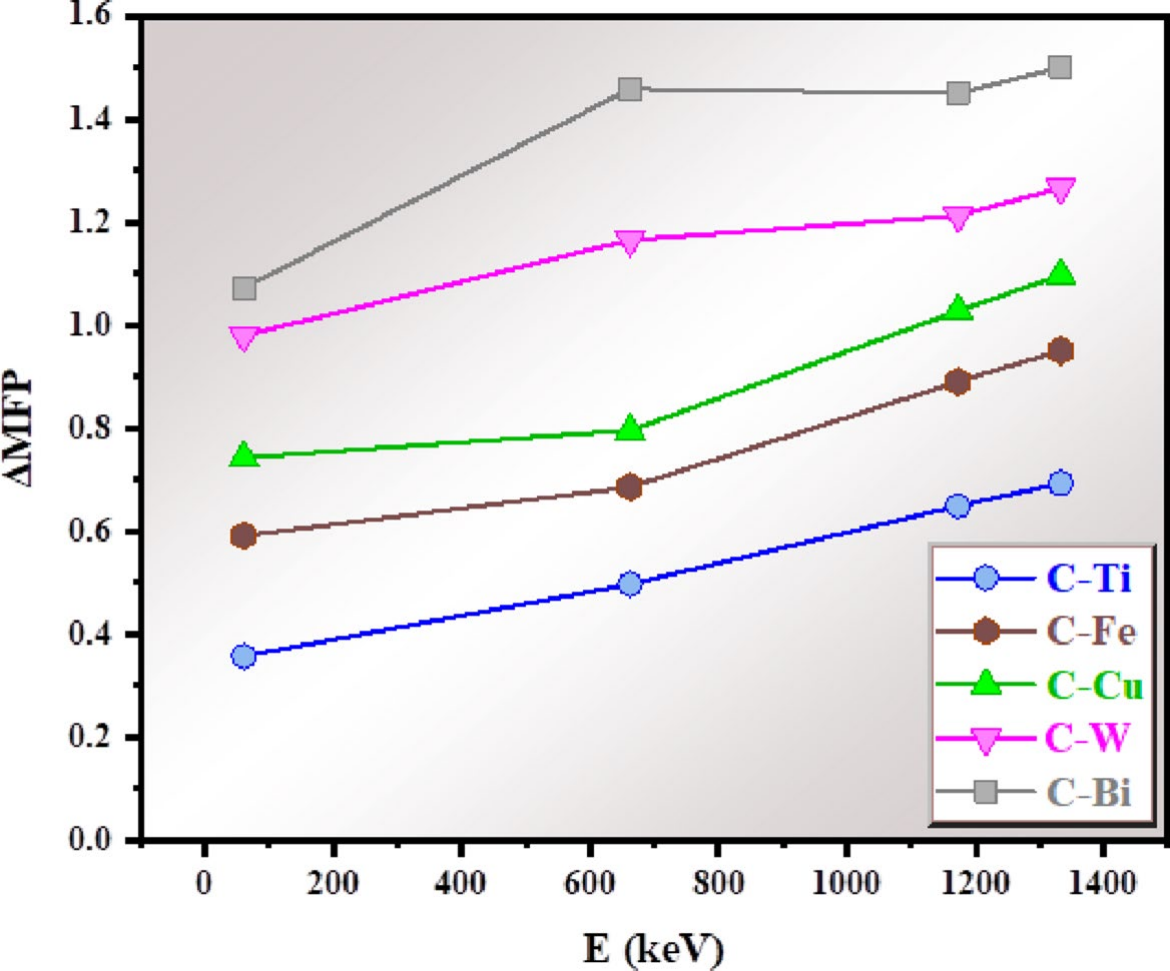
The difference in MFP (ΔMFP) between the reference clay and the modified clays.

Moreover, the difference in MFP (ΔMFP) between the reference clay and the clays with oxides is calculated and plotted in Fig. 13. This parameter examines the change in the MFP due to the introduction of TiO_2_, CuO, Fe_2_O_3_, WO_3_ and Bi_2_O_3_. The highest ΔMFP is observed for the clay with Bi_2_O_3_ followed by clay with WO_3_. This confirms the importance of using both heavy oxides (Bi_2_O_3_ and WO_3_) in radiation shielding technology. The lowest ΔMFP is observed for C-Ti due to the low density of TiO_2_ (ranging between 0.357 and 0.693), which means that this modified clay is close the reference sample and considerable improvement in the radiation shielding effectiveness is found for this sample. From Fig. 13 we can get the following conclusion: the inclusion of WO_3_ and Bi_2_O_3_ to the clay is more beneficial for gamma radiation shielding, while the relatively small ΔMFP values for C-Ti reflects the limited effect of low-density component in radiation shielding.

**Fig. 14:**
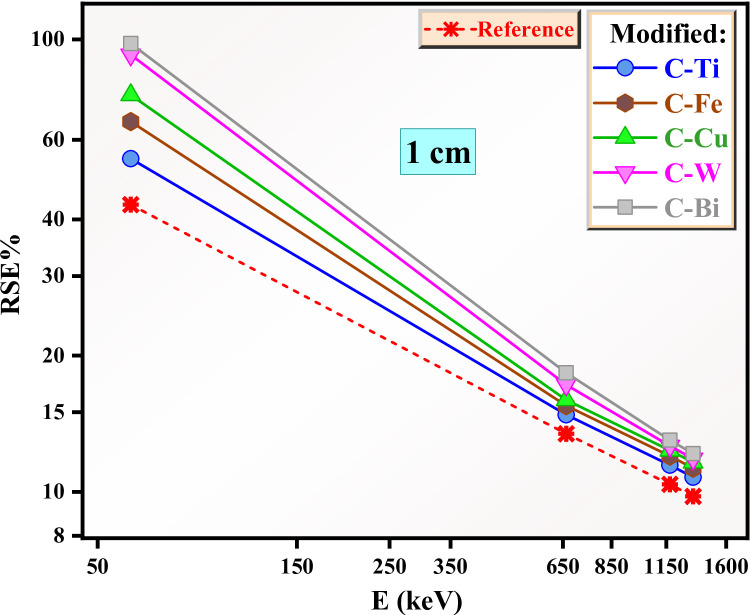
The RSE for the reference and modified clays with thickness 1 cm.

The RSE for the reference and modified clays with thickness 1 cm is plotted in Fig. 14. For the reference clay, the RSE is 43% at 0.059 MeV, improving to 55%, 66% and 75% for C-Ti, C-Fe and C-Cu respectively. The RSE for C-W and C-Bi at 0.059 MeV is approach to 98% suggests that almost gamma-radiations are absorbed by these two clays. Hence, C-W and C-Bi clays act as a superior barrier against the radiation with energy of 0.059 MeV. This is because tungsten and bismuth have high atomic number and the inclusion of these two high density compounds considerably improves the radiation shielding, thereby resulting in negligible transmission of the photons through C-W and C-Bi clays. At 0.662 MeV, the RSE for the reference is 13%, indicating the most photons are transmitted through this clay. The modified clays also show a relatively weak shielding performance at this energy, where RSE is around 16% for C-Ti, C-Fe and C-Fe, while it is ~ 18% for the clay with WO_3_ and Bi_2_O_3_. Figure 14 shows that a thickness of 1 cm for the reference and the modified clays are sufficient for low energy applications (less than 0.662 MeV), while for higher energy applications the thickness is insufficient to provide safe protection.

**Fig. 15:**
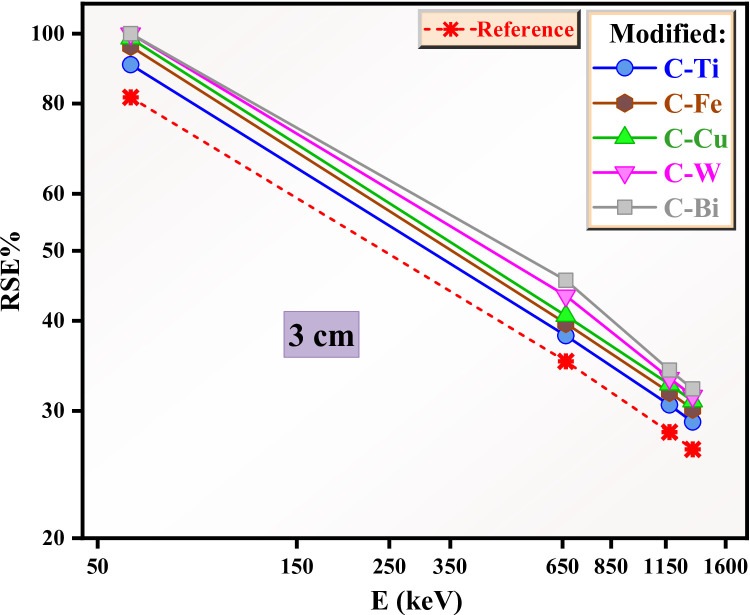
The RSE for the reference and modified clays with thickness 3 cm.

**Fig. 16:**
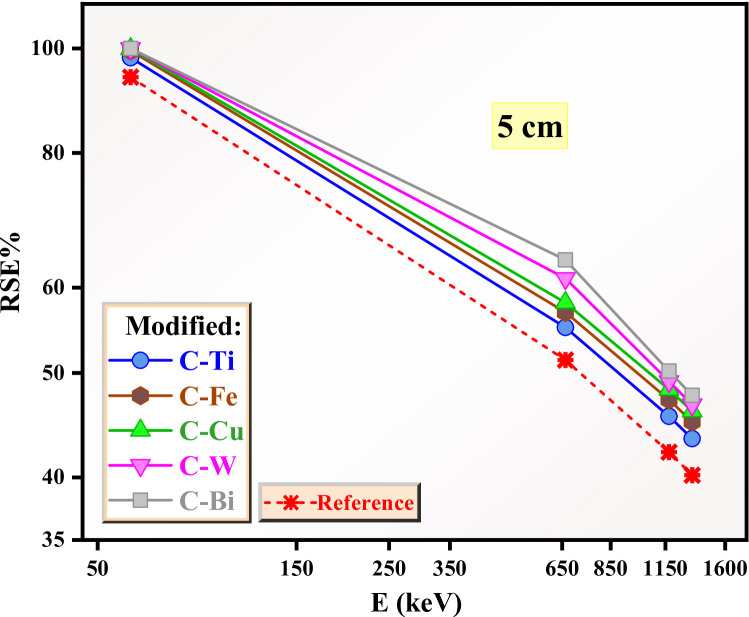
The RSE for the reference and modified clays with thickness 5 cm.

For this reason, we investigated the RSE for the reference and modified clays when the thickness increases to 3 and 5 cm (Figs. 15 and 16**)**. For the reference clay, the RSE is 82% and 95% in Figs. 15 and 16 at 0.059 MeV, suggesting improvement in the radiation protection of the reference sample with large thickness. The same result was observed for the modified clays at 0.059 MeV, suggesting full shielding for these clays against very low energy radiation. At 0.662 MeV, the RSE for the reference clay decreases to 35% (Fig. 15) and 51% (Fig. 16), indicating the importance of using thick clay for sufficient protection. The modified clays show good shielding performance especially those with thickness of 5 cm and with CuO, WO_3_ and Bi_2_O_3_, where the RSE is higher than 60%.

The present clay composites were compared by different works such as Ball clay and kaolin^56^, other different clay like Red-clay (S1), Green-clay (S2) and White-clay (S3)^57^, and different composites included Red-clay as a filler^58^, such as ERB-1 (40% Epoxy, 50% red-clay, 10% Bi_2_O_3_), ERB-2 (40% Epoxy, 40% red-clay, 20% Bi_2_O_3_) and ERB-3 (40% Epoxy, 30% red-clay, 30% Bi_2_O_3_). Based on the comparison and results shown in Table 2, the presented clay composites outperform those compared to them, confirming their suitability for use as shielding compounds at low and medium energies in appropriate applications.

**Table 2:** Comparison this study with different works.

Reference	Composite Code	Energy, keV	LAC, cm^− 1^	HVL, cm	TVL, cm
^56^	Ball Clay	609.31	0.157	4.41495	14.6661
	Kaolin		0.157	4.41495	14.6661
^57^	S1	661.71	0.152	4.56018	15.1486
	S2		0.153	4.53037	15.0496
	S3		0.152	4.56018	15.1486
^58^	ERB-1	661.71	0.145	4.78033	15.8799
	ERB-1		0.158	4.38701	14.5733
	ERB-1		0.174	3.9836	13.2332
Present Study	C-Cu	661.71	0.174	3.9836	13.2332
	C-W		0.189	3.66745	12.183
	C-Bi		0.202	3.43142	11.3989

## Conclusion

In present work, different metal oxides (TiO_2_, WO_3_, Fe_2_O_3_, Bi_2_O_3_, and CuO) are added to mixture of clay, gypsum, and marble waste. The shielding properties of these clay composites were measured at different energies (0.059, 0.662, 1.173, and 1.333 MeV). The results show increasing of LAC values with addition of different metal oxides. The sample containing Bi_2_O_3_ (C-Bi) exhibits highest LAC where it has LAC value of 3.864 cm^− 1^ compared to 0.564 cm^− 1^ for reference sample at 0.059 MeV and the LAC values decrease in order of C-Bi > C-W > C-Cu > C-Fe > C-Ti > C-0. On other hand, results of HVL values show weak attenuation ability of C-Ti sample where it has highest HVL value comparing to C-W and C-Bi samples which have lowest HVL values. Also, the results show the good efficiency of all clay samples with large thickness at lower energies where the RSE values are above 90% for all clay samples with thickness of 3 and 5 cm at 0.059 MeV. In addition, the C-W and C-Bi samples with lower thickness (1 cm) exhibit RSE values near to 95% at 0.059 MeV which makes it sufficient in lower energy shielding applications. On other hand, the results of thermogravimetric analysis show increasing of thermal stability for clay samples with addition of different metal oxides. While, it is noticed that compressive strength values decrease with lowest value for C-Bi sample which exhibits compressive strength value of 9.1 MPa compared to 18.41 MPa for reference sample.

## Data Availability

The data presented in this study are available on request from the corresponding author.
